# Carbon dioxide drives oviposition in *Helicoverpa armigera*

**DOI:** 10.1093/nsr/nwaf270

**Published:** 2025-07-10

**Authors:** Qiuyan Chen, Hetan Chang, Baiwei Ma, Mengbo Guo, Song Cao, Bin Li, Xiaoqing Wang, Bente G Berg, Xi Chu, Tiantao Zhang, Bill S Hansson, Yang Liu, Guirong Wang

**Affiliations:** State Key Laboratory for Biology of Plant Diseases and Insect Pests, Institute of Plant Protection, Chinese Academy of Agricultural Sciences, Beijing 100193, China; Shenzhen Branch, Guangdong Laboratory for Lingnan Modern Agriculture, Synthetic Biology Laboratory of the Ministry of Agriculture and Rural Affairs, Agricultural Genomics Institute at Shenzhen, Chinese Academy of Agricultural Sciences, Shenzhen 518120, China; Shenzhen Branch, Guangdong Laboratory for Lingnan Modern Agriculture, Synthetic Biology Laboratory of the Ministry of Agriculture and Rural Affairs, Agricultural Genomics Institute at Shenzhen, Chinese Academy of Agricultural Sciences, Shenzhen 518120, China; State Key Laboratory for Biology of Plant Diseases and Insect Pests, Institute of Plant Protection, Chinese Academy of Agricultural Sciences, Beijing 100193, China; Shenzhen Branch, Guangdong Laboratory for Lingnan Modern Agriculture, Synthetic Biology Laboratory of the Ministry of Agriculture and Rural Affairs, Agricultural Genomics Institute at Shenzhen, Chinese Academy of Agricultural Sciences, Shenzhen 518120, China; State Key Laboratory for Biology of Plant Diseases and Insect Pests, Institute of Plant Protection, Chinese Academy of Agricultural Sciences, Beijing 100193, China; State Key Laboratory for Biology of Plant Diseases and Insect Pests, Institute of Plant Protection, Chinese Academy of Agricultural Sciences, Beijing 100193, China; State Key Laboratory for Biology of Plant Diseases and Insect Pests, Institute of Plant Protection, Chinese Academy of Agricultural Sciences, Beijing 100193, China; Shenzhen Branch, Guangdong Laboratory for Lingnan Modern Agriculture, Synthetic Biology Laboratory of the Ministry of Agriculture and Rural Affairs, Agricultural Genomics Institute at Shenzhen, Chinese Academy of Agricultural Sciences, Shenzhen 518120, China; State Key Laboratory for Biology of Plant Diseases and Insect Pests, Institute of Plant Protection, Chinese Academy of Agricultural Sciences, Beijing 100193, China; Chemosensory Laboratory, Department of Psychology, Norwegian University of Science and Technology (NTNU), Trondheim 7491, Norway; Chemosensory Laboratory, Department of Psychology, Norwegian University of Science and Technology (NTNU), Trondheim 7491, Norway; State Key Laboratory for Biology of Plant Diseases and Insect Pests, Institute of Plant Protection, Chinese Academy of Agricultural Sciences, Beijing 100193, China; Department of Evolutionary Neuroethology, Max Planck Institute for Chemical Ecology, Jena 07745, Germany; State Key Laboratory for Biology of Plant Diseases and Insect Pests, Institute of Plant Protection, Chinese Academy of Agricultural Sciences, Beijing 100193, China; State Key Laboratory for Biology of Plant Diseases and Insect Pests, Institute of Plant Protection, Chinese Academy of Agricultural Sciences, Beijing 100193, China; Shenzhen Branch, Guangdong Laboratory for Lingnan Modern Agriculture, Synthetic Biology Laboratory of the Ministry of Agriculture and Rural Affairs, Agricultural Genomics Institute at Shenzhen, Chinese Academy of Agricultural Sciences, Shenzhen 518120, China

**Keywords:** carbon dioxide, *Helicoverpa armigera*, oviposition, plant, CO_2_ receptor

## Abstract

The levels of carbon dioxide (CO_2_), a powerful greenhouse gas, have risen dramatically over the past century, leading to widespread ecological effects on plants and animals alike, including insects that serve vital roles in many food webs. Elevated atmospheric CO_2_ is anticipated to affect insect biodiversity by influencing essential behaviors, although the mechanisms remain poorly understood. Here, we demonstrate that female *Helicoverpa armigera* use plant-emitted CO_2_ as a primary cue for egg-laying, showing a preference for younger leaves with higher CO_2_ gradients to enhance offspring survival. Elevated environmental CO_2_ disrupts this preference, reducing females’ attraction to optimal egg-laying sites. Employing genome editing tools, we assessed the CO_2_ receptors in this species and proved three gustatory receptors—HarmGR1, HarmGR2, and HarmGR3—that form a trimeric complex in the sensory neurons of the labial palp organ, essential for CO₂ detection. These neurons project to the labial pit organ glomerulus (LPOG) in the antennal lobe, which mediates CO_2_-responsive behavior. Genetic disruption of any of these receptors impairs CO_2_ sensing and alters oviposition behavior. Our findings underscore the essential role of CO_2_ in moth reproductive behavior and reveal that rising anthropogenic CO_2_ levels may have significant ecological and agricultural repercussions.

## INTRODUCTION

The accelerating pace of climate change is reshaping ecosystems globally, with significant implications for insect biodiversity [1–3]. Despite their success and remarkable abundance, which are due to their ability to colonize a wide range of ecological niches, insects are particularly vulnerable to shifts in temperature, precipitation, and atmospheric composition [4,5]. As global temperatures rise and weather patterns become increasingly erratic, the behaviors of insect species are being altered, which can have cascading effects on ecosystem dynamics and species interactions [6–8]. Understanding how climate change influences the life cycles, behaviors, and reproductive strategies of insects is thus crucial for preserving biodiversity and maintaining ecosystem health.

Among the principal contributors to climate change, carbon dioxide (CO_2_) plays a central role as a greenhouse gas, significantly affecting global climate patterns [9,10]. Atmospheric CO_2_ levels have risen dramatically from 278 ppm in 1750 to ∼420 ppm in 2023, largely due to human activities such as fossil fuel combustion and deforestation [11,12]. This increase has profound implications for global ecosystems, where insects represent the most abundant and diverse group of organisms [13–15]. They provide essential ecosystem services, including pollination, pest control, and nutrient cycling [16,17]. However, elevated CO_2_ levels are altering insect growth rates [6], population dynamics [8,18], and behaviors [19,20], leading to changes in complex ecological interactions and potential declines in biodiversity.

One of the most direct effects of elevated CO_2_ on insects is its influence on growth and development. While increased CO_2_ has been shown to augment both feeding and reproduction in some beetle species [6], other insect populations may decline under similar conditions [8,18]. More critically, elevated CO_2_ affects insect behaviors, particularly those related to reproduction, migration, and orientation [19,21,22]. For instance, host-seeking behaviors in mosquitoes are disrupted by higher CO_2_ concentrations, which can alter their ecological roles and interactions with other species [19].

A crucial yet often overlooked aspect of CO_2_’s influence on insect behavior is its role as a chemosensory cue [23]. CO_2_ can be directly detected by specialized gustatory receptors in many insect species, shaping their ecological adaptations and influencing key behaviors such as habitat selection and foraging strategies. For example, *Drosophila melanogaster* utilizes gustatory receptors GR21a and GR63a to detect elevated CO_2_ levels, triggering avoidance responses that can affect survival and reproductive success [24–27]. Similarly, gustatory receptors in mosquitoes, such as AaegGR1 and AaegGR3, are vital for locating potential hosts based on CO_2_ emissions [28,29]. In moths, the labial pit organ (LPO) is specialized for CO_2_ detection, where sensory neurons express three receptor genes critical for orienting behaviors in response to CO_2_ gradients [30–32]. These neurons target one specific glomerulus in the antennal lobe, the labial pit organ glomerulus (LPOG) [33]. The LPOG receives dendritic branches of CO_2_-specific projection neurons, which in turn target higher brain areas such as the mushroom bodies and the lateral horn [34].

The influence of rising CO_2_ on insect behavior also extends beyond direct chemosensory effects and encompasses broader ecological interactions. Elevated CO_2_ can alter plant chemistry, including the production of volatile organic compounds (VOCs) and secondary metabolites, which serve as crucial cues for insect herbivores when selecting host plants [35–37]. Such changes can affect host plant selection, feeding behavior, and overall herbivory rates. Elevated CO_2_ has been shown to alter growth and reproduction rates in several herbivorous insects including beetles and moths [6,38,39] and can disrupt existing ecological balances and influence community dynamics.

Despite significant advances in understanding how CO_2_ affects insect behaviors, much of the research has focused on host plant selection, feeding behavior, and habitat selection, leaving a critical gap in our knowledge regarding reproductive behaviors, particularly oviposition [23]. Oviposition behavior is essential for maintaining insect biodiversity, as it determines population dynamics and the successful establishment of insect communities within ecosystems [40,41].

To address this gap, we investigated the role of CO_2_ in the oviposition behavior of female *Helicoverpa armigera*, a major agricultural pest responsible for significant crop losses worldwide. We show that female moths preferentially lay their eggs on young plant leaves characterized by higher CO_2_ concentrations, thereby enhancing offspring survival and fitness due to the correlation between CO_2_-rich environments and favorable nutrient availability. Under projected atmospheric conditions, with CO_2_ levels reaching 1000 ppm by 2100 [42,43], this oviposition preference will be disrupted, potentially leading to unpredictable shifts in insect biodiversity and community dynamics. To elucidate the molecular basis of this behavior, we identified three gustatory receptors—HarmGR1, HarmGR2, and HarmGR3—expressed in the labial palps that mediate CO_2_ detection in *H. armigera*. Disruption of any of these receptors significantly impaired the moths’ ability to detect CO_2_, resulting in altered oviposition behavior. These results underscore the critical role of CO_2_ as a sensory cue in insect reproduction and highlight the potential for climate change to influence insect biodiversity through changes in CO_2_-driven oviposition behaviors.

## RESULTS

### Oviposition on young leaves increases offspring fitness in *H. armigera*

**Figure 1.**
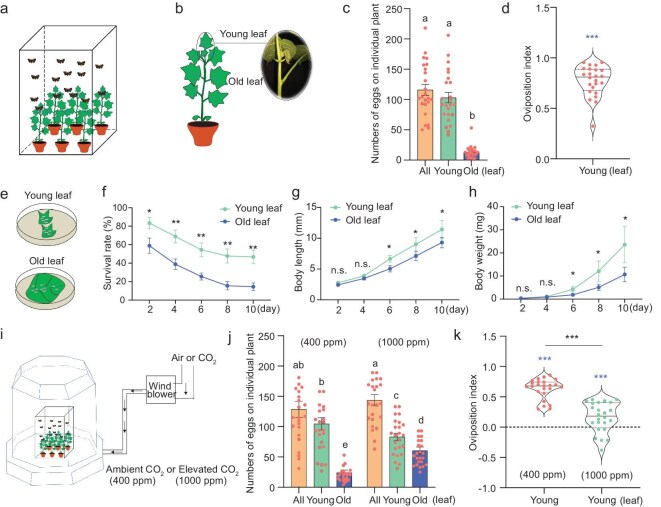
Atmospheric CO_2_ elevation impacts *Helicoverpa armigera* reproductive capacity by disrupting oviposition behavior. (a) Schematic representation of oviposition experiments conducted in a semi-natural environment cage. Details of the experiments are described in the Materials and Methods. (b) Photograph showing a female laying eggs on a cotton plant in the leaf stage, with encircled areas indicating numerous eggs deposited on young leaves. (c) Number of eggs deposited on the entire plant, as well as on young and old leaves. (d) Oviposition index of female moths laying eggs on young versus old leaves, calculated as (Y − O)/(O + Y), where Y is the number of eggs deposited on young leaves and O is the number of eggs deposited on old leaves. (e) Drawing depicting the benefits of feeding on young leaves for the growth and development of offspring compared to old leaves. (f–h) Quantification of survival rates (f), body length (g), and body weight (h) of larvae fed on young versus old leaves. (i) Schematic of oviposition preference tests conducted in two open-top chambers (OTCs) with and without CO_2_ exposure. (j) Quantification of the number of eggs deposited on all leaves, young leaves, and old leaves of individual plants in the two OTCs with and without CO_2_ exposure. (k) Oviposition index of mated females on young leaves of cotton plants in the two OTCs with and without CO_2_ exposure. In (c) and (j), lowercase letters indicate statistical significance at *p* < 0.05 (Duncan's multiple range test, *n* = 24). In (d) and (k), blue asterisks indicate statistical significance of the index mean from ‘0’ at *p* < 0.001 (paired two-tailed Student's *t*-test, *n* = 24). Black asterisks indicate statistical significance between the two OTCs at *p* < 0.001 (unpaired two-tailed Student's *t*-test, *n* = 24). The violin plots display data distributions with inner lines representing the median (center line) and first/third quartiles (box edges). In (f–h), n.s. indicates *p* > 0.05; * indicates *p* < 0.05; ** indicates *p* < 0.01 (unpaired two-tailed Student's *t*-test, *n* = 9). All data are presented as mean ± SEM.

Before investigating the cues driving oviposition in *H. armigera*, we aimed to characterize their oviposition behavior. Numerous studies indicate that female moths preferentially lay eggs on young and tender leaves compared to older, mature leaves [44–46]. We utilized cotton plants at the stage of eight true leaves as substrates to examine female oviposition choice in a caged semi-natural environment (Fig. 1a). In this bioassay, we found that females laid most eggs on leaves, with only minimal oviposition occurring on other plant parts. We therefore focused exclusively on quantifying egg deposition on cotton leaves in our analysis. We observed a significantly higher number of eggs on young leaves compared to old ones, confirming that *H. armigera* preferentially oviposits on younger leaves of host plants (Fig. 1b–d, and Fig. S1a). To assess the ecological significance of this oviposition preference, we recorded the survival rate, body length, and body weight of newly hatched larvae feeding on young versus old leaves (Fig. 1e). The survival rate of larvae feeding on young leaves was significantly higher than that of larvae on old leaves (Fig. 1f). In terms of body length and weight, no significant differences were observed during the first four days; however, from day six onward, larvae feeding on young leaves exhibited significantly greater body lengths and weights compared to those feeding on old leaves (Fig. 1g and h). These findings indicate that the oviposition strategy of *H. armigera*, laying eggs on young leaves, is adaptive and beneficial for the growth and development of the offspring.

### Elevated atmospheric CO_2_ disrupts oviposition preference in *H. armigera*

Emerging evidence indicates that elevated atmospheric CO_2_ concentrations adversely affect the oviposition behavior of insects on host plants [47,48]. To study the oviposition preferences under increased CO_2_ levels, we simulated future atmospheric conditions, projecting a rise from the current concentration of ∼400 ppm to 1000 ppm by the year 2100 [42,43], a level historically correlated with mass extinction events [49]. We examined the oviposition behavior of *H. armigera* in open-top chambers (OTCs) under two CO_2_ conditions: 1000 ppm (elevated) and 400 ppm (ambient control) (Fig. 1i). Consistent with previous observations in semi-natural environments, we found that under ambient conditions (400 ppm CO_2_), mated *H. armigera* females deposited a significantly greater number of eggs on young leaves compared to older ones (Fig. 1j and Fig. S2a). In contrast, at elevated CO_2_ levels (1000 ppm), we observed a significant decrease in the number of eggs on young leaves, accompanied by a marked increase in egg counts on older leaves (Fig. 1j and Fig. S2b). As a result, the oviposition index for young leaves decreased significantly from 0.63 ± 0.035 at 400 ppm CO_2_ to 0.16 ± 0.050 at 1000 ppm CO_2_. Collectively, these findings demonstrate that increased atmospheric CO_2_ levels significantly impair the oviposition preference of *H. armigera* for young leaves, suggesting that moths rely on plant-emitted CO_2_ as a key cue for identifying optimal oviposition sites.

### Young leaves emit higher concentrations of CO_2_ in comparison to old leaves

**Figure 2.**
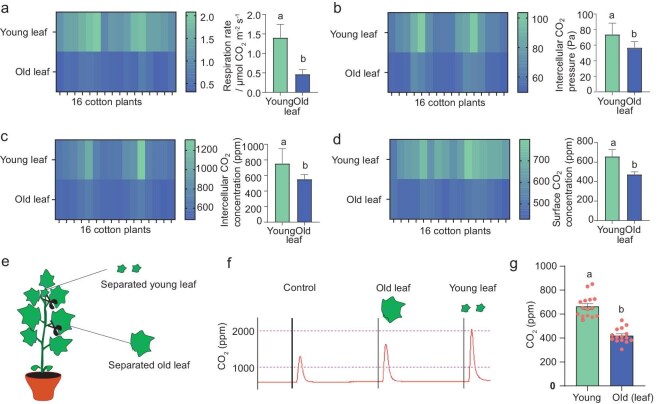
Young leaves emit more CO_2_ than old leaves. (a–d) Comparison of respiration rate (a), intercellular CO_2_ pressure (b), intercellular CO_2_ concentration (c), and surface CO_2_ concentration (d) in young versus old leaves on an intact cotton plant (*ON plant* condition). Data are presented as mean ± SEM, with lowercase letters indicating statistical significance at *p* < 0.05 (paired two-tailed Student's *t*-test, *n* = 16). (e) Schematic representation of young versus old leaves collected from the plant (*OFF plant* condition) for CO_2_ emission measurements. (f) A representative reading illustrating the amount of CO_2_ produced by young leaves versus old leaves collected from the plant. (g) Quantification of CO_2_ levels produced by young and old leaves. Data are presented as mean ± SEM, with lowercase letters specifying statistical significance at *p* < 0.05 (paired two-tailed Student's *t*-test, *n* = 15).

It has been well-established that young plant tissues emit higher levels of CO_2_ due to increased metabolic activity [50,51]. This condition could constitute a crucial cue for female *H. armigera* when searching for optimal oviposition sites. To test this hypothesis, we first measured CO_2_ emissions from young and old cotton leaves using a commercial leaf photosynthesis system (Fig. S3a). We assessed the respiration rate (Fig. 2a), intercellular CO_2_ pressure (Fig. 2b), intercellular CO_2_ concentration (Fig. 2c), and surface CO_2_ concentration (Fig. 2d), finding that, across all parameters, young leaves exhibited significantly higher CO_2_ emissions compared to older leaves (Table S1). To confirm these findings across different plant samples, we collected young and old leaves from individual plants and measured their CO_2_ emission levels using a Li-7000 CO_2_ analyzer (LI-COR; Fig. 2e and Fig. S3b). By controlling for leaf weight, we placed equivalent masses of young and old leaves into a chamber connected to the analyzer. Again, we found that the young leaves emitted significantly more CO_2_ than older leaves (Fig. 2f–g), confirming the initial results. Altogether, our findings reveal that young leaves emit ∼200 ppm more CO_2_ than older leaves, potentially providing a critical cue for mated *H. armigera* females in selecting optimal oviposition sites.

### CO_2_ induce female moth oviposition behaviors

**Figure 3.**
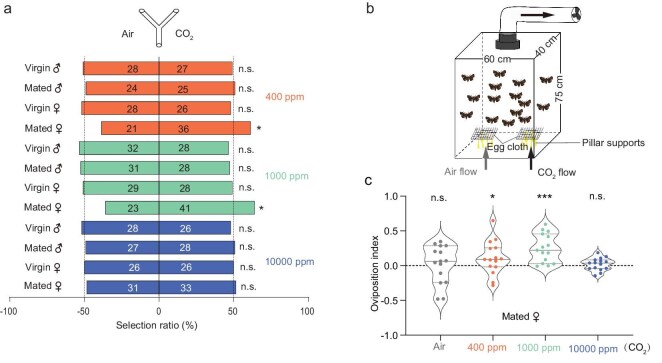
CO_2_ is a major cue inducing oviposition behavior in mated female *H. armigera*. (a) Behavioral responses of virgin and mated *H. armigera* to CO_2_ concentrations (400, 1000, and 10 000 ppm) in a Y-tube olfactometer. n.s. indicates *p* > 0.05; * indicates *p* < 0.05 (chi-square test, *n* = 60–80). (b) Schematic representation of the dual-choice oviposition experiments. (c) Quantification of the oviposition index from mated *H. armigera* females in response to CO_2_ at different concentrations. n.s. indicates *p* > 0.05; * indicates *p* < 0.05; *** indicates *p* < 0.001 (paired two-tailed Student's *t*-test, *n* = 15). The violin plots display data distributions with inner lines representing the median (center line) and first/third quartiles (box edges).

To determine whether CO_2_ really acts as a key chemical cue and induces oviposition behavior in *H. armigera*, we first tested the behavioral response of adult moths to CO_2_ signals. Using a Y-tube olfactometer, we assessed the responses of virgin and mated moths of both sexes to various concentrations of CO_2_ (400 ppm, 1000 ppm, and 10 000 ppm) compared to CO_2_-free synthetic air. Our results showed that only mated females were attracted to 400 ppm and 1000 ppm CO_2_ (Fig. 3a), with this attraction diminishing at the higher concentration of 10 000 ppm. In contrast, virgin females and both mated and virgin males did not exhibit any significant response to any of the CO_2_ concentrations tested. Given that only mated females exhibited attraction to CO_2_, we conducted a two-choice oviposition assay in oviposition cages to further examine the moth egg-laying preferences (Fig. 3b). Consistent with the Y-tube olfactometer results, mated females showed a significant preference for laying eggs in the presence of 400 ppm and 1000 ppm CO_2_ compared to synthetic air, while no preference was observed at 10 000 ppm concentration (Fig. 3c).

### Three gustatory receptors are essential for CO_2_ detection in moths

To investigate how *H. armigera* detects CO_2_, we focused on three putative gustatory receptor genes: *HarmGR1, HarmGR2*, and *HarmGR3* (Fig. S4a), which are localized in a specialized organ, the labial palp organ (LPO), known to be involved in CO_2_ detection in other moth species [31]. To confirm the expression patterns of these receptors, we performed quantitative RT-PCR (qRT-PCR) analyses on eight different organs from both female and male *H. armigera*. Consistent with previous reports, we found that all three genes were predominantly expressed in the labial palps of both sexes (Fig. S4b), while expression levels in other organs were notably lower, underscoring the crucial role of these receptors in CO_2_ detection.

Next, we aimed to elucidate how these receptors detect CO_2_. Although prior work had functionally expressed these receptors in an *in vitro* system and confirmed that HarmGR1 and HarmGR3 are indispensable and sufficient for CO_2_ response [31], *in vivo* verification was still lacking. To further investigate the *in vivo* functional roles of these GR receptors in CO_2_ detection, we employed CRISPR/Cas9 technology to individually knock out each receptor. Using a single guide RNA (sgRNA) targeting the first exon of each gene, we induced distinct deletions and insertions (Fig. S5a–c), resulting in specific premature stop codons for all three genes.

**Figure 4.**
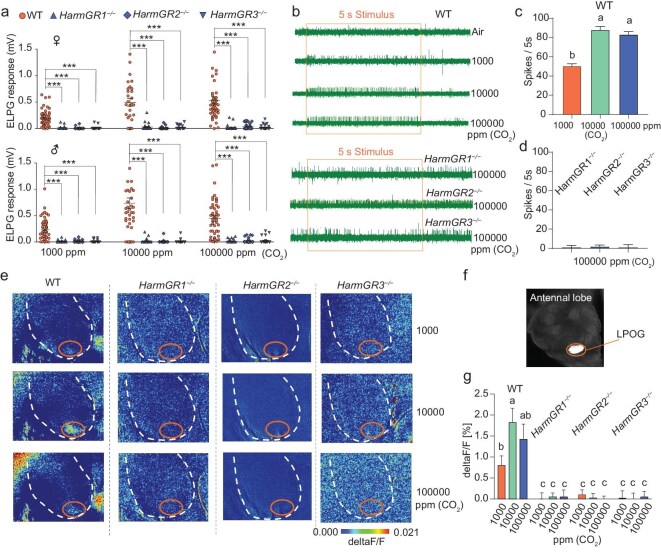
*HarmGR1, HarmGR2*, and *HarmGR3* are indispensable for CO_2_ detection in *H. armigera*. (a) Electrolabialpalpography (ELPG) responses to CO_2_ in wild-type (WT), *HarmGR1^−/−^, HarmGR2^−/−^*, and *HarmGR3^−/−^* mutants of *H. armigera*. Data are presented as mean ± SEM, with *** indicating *p* < 0.001 (unpaired two-tailed Student's *t*-test, *n* = 30–50). (b) Representative spike traces from single sensillum recordings (SSR) of LPO sensory neurons in WT, *HarmGR1^−/−^, HarmGR2^−/−^*, and *HarmGR3*^−/−^ mutants in response to CO_2_ at different concentrations. (c) Quantification of SSR responses in WT to CO_2_ at different concentrations. (d) Quantification of SSR responses in the three mutants to CO_2_ at 100 000 ppm. In (c) and (d), data are presented as mean ± SEM, with lowercase letters indicating statistical significance at *p* < 0.05 (unpaired two-tailed Student's *t*-test, *n* = 13–15). (e) Calcium responses among antennal lobe glomeruli of WT and mutant moths to CO_2_ at different concentrations. (f) Confocal image highlighting the location of the LPOG. (g) Quantification of calcium responses of LPOG in WT and mutant moths to CO_2_ at different concentrations. Data are presented as mean ± SEM, with lowercase letters indicating statistical significance at *p* < 0.05 (Duncan's multiple range test, *n* = 4).

Next, we used these mutant lines to examine the electrophysiological and calcium imaging responses of *H. armigera* to CO_2_, thereby validating the significance of these genes in mediating CO_2_ detection. We first performed electrolabialpalpography (ELPG) recordings from the whole labial palp. Since our above-mentioned behavioral tests on wild-type (WT) moths demonstrated that only mated females were attracted to CO_2_, whereas virgin females and mated/virgin males showed no significant response (Fig. 3a), we performed ELPG on both virgin and mated WT moths of both sexes in a pre-test to determine whether they could detect CO_2_. The results showed that there was no statistically significant difference in CO_2_ response between virgin and mated moths in either sex (Fig. S6a). We then conducted ELPG recordings on mutant moths and compared these with corresponding recordings from virgin WT moths. Our results indicated that the *HarmGR1, HarmGR2*, and *HarmGR3* knockout homozygous moths exhibited no response to any of the tested CO_2_ concentrations (Fig. 4a and Fig. S6b and c), while WT moths exhibited clear voltage deflections in response to CO_2_ stimuli at all tested concentrations (1000 ppm, 10 000 ppm, and 100 000 ppm; see Fig. 4a and Fig. S6b and c). To further explore whether the labial palp was the unique organ detecting CO_2_, we also assessed the potential response to CO_2_ in the main olfactory pathway—the antennae. We performed electroantennography (EAG) and found that both WT and mutant *H. armigera* antennae showed no response to CO_2_ stimulation (Fig. S6d and e), confirming that the antennae are not directly involved in CO_2_ detection.

Next, we examined CO_2_ responses at the single-neuron level using single sensillum recording techniques on sensilla in the LPO. In WT moths, we recorded from a total of 27 sensilla in the labial palp from eight females and five males. Among these, 15 exhibited an increased firing rate in response to CO_2_ stimulation, with notably stronger responses to 10 000 ppm and 100 000 ppm CO_2_ compared to 1000 ppm CO_2_ (Fig. 4b and c). We conducted similar recordings in the mutants, collecting data from 13 sensilla in *HarmGR1^−/−^*, 13 in *HarmGR2^−/−^*, and 15 in *HarmGR3^−/−^*, and found that none of the recorded neurons in these mutants displayed changes in firing frequency during CO_2_ stimulation (Fig. 4b and d).

Finally, we employed calcium imaging techniques to visualize the calcium dynamics of OSN terminals in the glomerular population of the antennal lobe. We found that WT moths exhibited significant responses to CO_2_ in the LPO glomerulus (LPOG), which is specifically targeted by sensory neurons originating from the LPO [52], upon stimulation with CO_2_ (Fig. 4e). More importantly, the LPOG was the only glomerulus that showed responses to different CO_2_ concentrations at the selected focal level. In contrast, the *HarmGR1^−/−^, HarmGR2^−/−^*, and *HarmGR3^−/−^* moths did not exhibit any responses in the LPOG during CO_2_ stimulation (Fig. 4f and g).

Collectively, the results from ELPG and single sensillum recordings, and calcium imaging experiments indicate that all three GRs are essential for CO_2_ detection in *H. armigera*.

### All three receptors are indispensable for CO_2_-induced oviposition behavior

**Figure 5.**
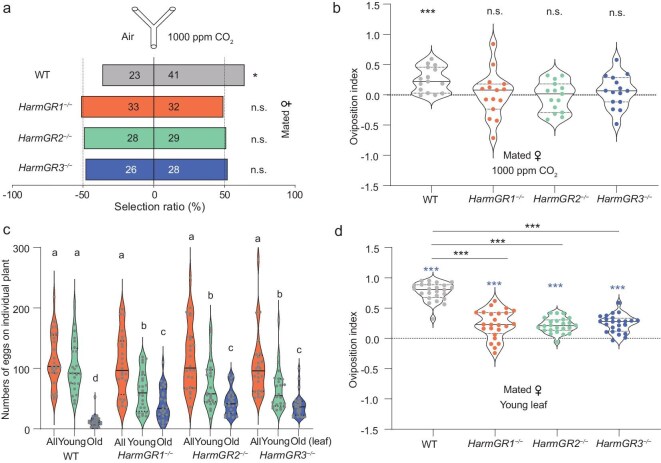
*HarmGR1, HarmGR2*, and *HarmGR3* are indispensable for CO_2_-induced oviposition behaviors in *H. armigera*. (a) Behavioral responses of mated females to 1000 ppm CO_2_ in wild-type (WT), *HarmGR1^−/−^, HarmGR2^−/−^*, and *HarmGR3^−/−^* mutants, measured in a Y-tube olfactometer. n.s. indicates *p* > 0.05; * indicates *p* < 0.05 (chi-square test, *n* = 70–80). (b) Oviposition preferences for CO_2_ in WT, *HarmGR1^−/−^, HarmGR2^−/−^*, and *HarmGR3^−/−^* mutants. The violin plots display data distributions with inner lines representing the median (center line) and first/third quartiles (box edges), with n.s. indicating *p* > 0.05; *** indicating *p* < 0.001 (paired two-tailed Student's *t*-test, *n* = 15). (c) Number of eggs deposited on the entire plant, as well as on young and old leaves in WT, *HarmGR1^−/−^, HarmGR2^−/−^*, and *HarmGR3*^−/−^ mutants. Data are presented as mean ± SEM; lowercase letters indicate statistical significance at *p* < 0.05 (Duncan's multiple range test, *n* = 24). (d) Oviposition preferences of WT, *HarmGR1^−/−^, HarmGR2^−/−^*, and *HarmGR3*^−/−^ for young leaves in a semi-natural environment. Blue asterisks indicate statistical significance between the index mean and ‘0’ at *p* < 0.001 (paired two-tailed Student's *t*-test, *n* = 24). Black asterisks indicate statistical significance between each mutant strain and WT at *p* < 0.001 (unpaired two-tailed Student's *t*-test, *n* = 24). The violin plots display data distributions with inner lines representing the median (center line) and first/third quartiles (box edges).

To investigate whether impaired CO_2_ detection affects the behavioral response in the three receptor mutants of *H. armigera*, we subjected knockout mutants (*HarmGR1^−/−^, HarmGR2^−/−^*, and *HarmGR3^−/−^*) alongside WT mated females to a two-choice Y tube olfactometer to assess the behavioral response to CO_2_. In all three receptor knockouts we observed a complete abolition of CO_2_ attraction, contrasting sharply with the clear attraction responses exhibited by WT females (Fig. 5a). Next, we examined the oviposition index of WT and mutant mated females to 1000 ppm CO_2_ in the oviposition cage, as described earlier (Fig. 3b). All three mutant females exhibited a total loss of egg-laying preferences for CO_2_ compared to their WT counterparts (Fig. 5b).

To mimic natural oviposition scenarios, we next used plants as substrates within a semi-natural environment (Fig. 1a). We quantified the total number of eggs laid on the entire plant and their distribution across different parts of the cotton plant. Notably, while the overall egg count did not differ between WT and mutant females, the preference to oviposit on young leaves was significantly reduced in the mutants compared to the WT moths (Fig. 5c and Fig. S1a–d). With impaired CO_2_ detection, the female preference to oviposit on younger leaves was significantly weakened (Fig. 5d and Fig. S1a–d). Collectively, these findings further support the notion that CO_2_-induced oviposition preference relies on the function of all three receptors.

## DISCUSSION

CO_2_ is a well-known greenhouse gas and its effect on our climate is reported in the world news on a daily basis. For insects, CO_2_ level dynamics carry significant information and sensitive detection systems have evolved to allow this information to be captured [23]. As anthropogenic activities continue to contribute to rising atmospheric CO_2_ levels, insects face significant ecological challenges. Studies have reported that elevated CO_2_ levels alter insect feeding behavior [53,54], adversely impact development [55], and disrupt population dynamics [56]. However, in parallel, insects evolve new behavioral responses to these environmental changes. For instance, increased CO_2_ may promote feeding behavior [57] and facilitate social interactions [58,59].

While the importance of CO_2_ for host-seeking in blood feeding insects has been intensely studied [60,61], its effects on oviposition in insect species remain less understood, only a few studies document its effects in moth species such as *Cactoblastis cactorum* [47,62], *Manduca sexta* [48,63,64], and *Hyphantria cunea* [65]. For instance, in *M. sexta* CO_2_ is a positive cue for flower location [63,64], while it seems to have a negative impact on female egg deposition [48]. Some effects on egg deposition have also been registered in other moth species [47], but a direct effect on oviposition site choice remained to be investigated.

Here, we demonstrate that mated *H. armigera* females use plant-emitted CO_2_ as a positive cue to identify optimal oviposition sites. *H. armigera* females are guided by a significant CO_2_ emission gradient between young and older leaves, favoring the former for oviposition—a choice beneficial for the development of newly hatched larvae. This link between oviposition site selection and offspring development underscores an ecological strategy that enhances reproductive fitness [66], consistent with observations in other Heliothinae species that similarly prefer young plant parts for egg-laying [44–46]. Another interesting observation from our data revealed that mated/virgin males and virgin females—in contrast to mated ones—exhibited no CO_2_ preference. However, electrophysiological recordings of ELPG responses showed that both virgin and mated moths of both sexes were able to detect CO_2_ to the same extent, as similar ELPG responses were recorded. This finding prompts exploration of whether CO_2_ perception undergoes central modulation based on age, developmental stage, or hormonal status of individual insects. Such a phenomenon has e.g. been reported for sensing 11-cis-vaccenyl acetate (cVA) in *Drosophila*, where cVA detection is mediated by Or67d in both sexes, but evokes sex-specific behaviors—promoting aggression in males and modulating mating receptivity in females. The behavioral responses of *H. armigera* to CO₂ are thus likely not only to be regulated by peripheral detection but also involve signal integration and processing within the central nervous system [67–69].

Beyond its role in *H. armigera* ecology, CO_2_ has been widely exploited as a behavioral cue for pest management. For instance, studies have documented its application in sustainable control strategies for adult mosquitoes and subterranean pests, which rely on CO_2_ for host or food location [70–74]. Building on these precedents, our findings suggest that CO_2_ could similarly serve as the basis for novel attract-and-kill strategies targeting *H. armigera*, leveraging the role of CO_2_ as an oviposition attractant to disrupt reproductive success.

Even though significantly weakened, the oviposition preference for young leaves persists even in mutants lacking CO_2_ detection capabilities. This suggests that, while CO_2_ facilitates oviposition preferences, other sensory modalities—including olfaction, gustation, mechanosensation, and vision—also play a critical role in site selection [75,76]. Multiple sensory cues thus very likely contribute to oviposition behavior, a concept supported by previous studies on e.g. fruit flies and mosquitoes [77–80].

The impact of elevated environmental levels of CO_2_ on insects is complex and multifaceted. As the most abundant and diverse group of organisms, insects play vital roles in pollination, pest control, nutrient cycling, and as food sources for higher trophic levels [16,17]. Studies have shown that increased atmospheric CO_2_ concentrations affect insect population dynamics, primarily by altering reproductive behaviors [21]. High, fluctuating CO_2_ levels have been shown to decrease the number of offspring in moths like *C. cactorum* and *M. sexta* due to impaired host plant location [47,48]. In our study, we show that elevated CO_2_ levels directly influenced oviposition preferences in *H. armigera*. A shift in oviposition behavior, away from nutritionally favorable young leaves, could lead to unforeseen consequences for egg hatching success, potentially impacting insect biodiversity and ecosystem dynamics.

The detection of CO_2_ in insects involves specialized receptors and neural circuits evolved for diverse ecological functions. In *D. melanogaster*, CO_2_ detection is mediated by two gustatory receptors, DmelGR21a (GR1 sub-group) and DmelGR63a (GR3 sub-group), which form a heterodimeric complex that triggers avoidance behaviors—likely an adaptation to evade environments with high CO_2_ concentrations [24,25]. In contrast, many other insects, including mosquitoes, moths, and beetles, possess an additional CO_2_ receptor from the GR2 sub-group, forming a heterotrimeric complex for more nuanced CO_2_ detection [81]. In *H. armigera*, however, the roles of the different CO_2_ receptors have been controversial. Prior *in vitro* studies have yielded conflicting results. Xu and Anderson [82] reported that only HarmGR3 was activated by NaHCO_3_ when expressed in insect Sf9 cells, suggesting a primary role for this receptor. Conversely, Ning *et al*. [31] found that both HarmGR1 and HarmGR3 were essential for CO_2_ sensing in *Xenopus laevis* oocytes, indicating that multiple receptors contribute to detection. To resolve these discrepancies and clarify the interactions *in vivo*, we utilized CRISPR/Cas9 genome editing to generate null mutations for each of the three CO_2_ receptors in *H. armigera*. Electrophysiological recordings from the labial palps of mutant adults showed that the loss of any single receptor abolished neural responses to CO_2_. Behavioral assays further demonstrated that these mutants no longer exhibited attraction to elevated CO_2_ levels during oviposition site selection. These findings show that in their natural location all three receptors—HarmGR1, HarmGR2, and HarmGR3—are essential for CO_2_ detection in adult *H. armigera*. The co-expression arrangement of three receptors in CO_2_-detecting sensory neurons in *H. armigera* closely aligns with the co-expression of two CO_2_ receptors in *D. melanogaster*, where DmelGR21a and DmelGR63a are essential for CO_2_ detection [24,25]. However, the prerequisite of three functional receptors is somewhat different to previous results obtained in mosquitoes and beetles. In *Aedes aegypti* and *Anopheles coluzzii*, GR1 and GR3 are absolutely required to maintain *in vivo* CO_2_ sensitivity, while, in contrast, knockdown of GR2 resulted in insects still able to respond to CO_2_ stimuli [29,83]. Nevertheless, DvvGR2 is specifically required for larval responses to CO_2_ in *Diabrotica virgifera virgifera* [84]. The requirement of a trimeric receptor complex may enhance *H. armigera*’s ability to detect and respond to varying CO_2_ concentrations, providing adaptive advantages for oviposition site selection and offspring survival. Furthermore, the evolutionary conservation of GR2, alongside GR1 and GR3, across diverse insect species and families suggests that a trimeric configuration might offer selective benefits in several different ecological contexts.

The functional identification of CO_2_ receptors in *H. armigera* could potentially serve as targets for innovative pest management strategies. By using innovative approaches such as RNA interference, (RNAi)-based gene silencing could be developed to impair the insect's ability to detect CO_2_ gradients, thereby reducing oviposition accuracy and reproductive success. Similar approaches have been successful in the management of other pest insects: in *A. aegypti* and *D. virgifera virgifera*, silencing of CO_2_-sensitive receptors significantly reduced host-seeking behavior [29,84], while in the model insect *D. melanogaster*, disruption of CO_2_-sensitive neurons impaired avoidance responses [85].

**Figure 6.**
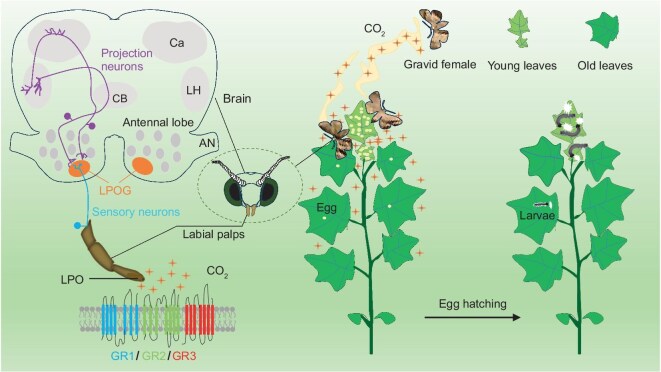
Schematic representation of the sensory mechanisms underlying CO₂-induced oviposition behavior in *H. armigera*. Abbreviations: LPO, labial pit organ; LPOG, labial pit organ glomerulus; CB, central body; Ca, calyx of the mushroom body; LH, lateral horn; AN, antennal nerve.

In conclusion, our findings underscore the critical role of CO_2_ as a cue in insect reproductive behavior and reveal the molecular and neural basis of CO_2_ detection in *H. armigera* (Fig. 6). By demonstrating how rising atmospheric CO_2_ disrupts this reproductive behavior, we highlight the broader ecological consequences of climate change on insect biodiversity and ecosystem dynamics. Understanding the ecological and evolutionary dynamics of CO_2_ detection is vital for predicting insect responses to global environmental change. Our study underscores the urgent need to mitigate anthropogenic CO_2_ emissions to preserve ecosystem stability.

## MATERIALS AND METHODS

The detailed materials and methods are available as a Supplementary file.

## Supplementary Material

nwaf270_Supplemental_File

## DATA AND MATERIALS AVAILABILITY

All data are available in the main text or the supplementary file. The successfully cloned GR ORF sequences in this study have been submitted to NCBI, and their respective GenBank accession numbers are OR734304, OR734305, and OR734306.
