# Supplementary material for: Carbon dioxide drives oviposition in Helicoverpa armigera
Source: Natl Sci Rev. 2025 Jul 10;12(12):nwaf270. doi: 10.1093/nsr/nwaf270 (PMC12693517; doi:10.1093/nsr/nwaf270)
Supplement: nwaf270_Supplemental_File [file nwaf270_supplemental_file.docx]

Supplementary information for

**Carbon dioxide drives oviposition in *Helicoverpa* *armigera***

Qiuyan Chen^1,2^, Hetan Chang^2^, Baiwei Ma^1^, Mengbo Guo^1^, Song Cao^1^, Bin Li^1^, Xiaoqing Wang^1^, Bente G. Berg^3^, Xi Chu^3^, Tiantao Zhang^1^, Bill S. Hansson^4^, Yang Liu^1^*, Guirong Wang^1,2^*

^1^ State Key Laboratory for Biology of Plant Diseases and Insect Pests, Institute of Plant Protection, Chinese Academy of Agricultural Sciences, Beijing 100193, China.

^2^ Shenzhen Branch, Guangdong Laboratory for Lingnan Modern Agriculture, Synthetic Biology Laboratory of the Ministry of Agriculture and Rural Affairs, Agricultural Genomics Institute at Shenzhen, Chinese Academy of Agricultural Sciences, Shenzhen 518120, China.

^3^ Chemosensory Laboratory, Department of Psychology, Norwegian University of Science and Technology (NTNU), Trondheim, Norway.

^4^ Department of Evolutionary Neuroethology, Max Planck Institute for Chemical Ecology, 07745 Jena, Germany.

*Corresponding Authors: Yang Liu and Guirong Wang. Emails: yangliu@ippcaas.cn; wangguirong@caas.cn

**This PDF file includes:**

Materials and Methods

References cited in the materials and methods

Figs. S1 to S7

Tables S1 to S2

**Materials and Methods**

**Insects and plants**

The *H*. *armigera* colony was cultivated at the Institute of Plant Protection, Chinese Academy of Agricultural Sciences in Beijing, China. Insect rearing conditions included a 14:10 (light:dark) photoperiod at a constant temperature of 27 ± 1°C, with a relative humidity maintained at 65 ± 5%. Larvae were raised on an artificial diet, and pupae were collectively housed in a gauze cage prior to eclosion. Adult moths were segregated by sex, placed in individual glass tubes, and provided with a daily diet of 10 % (w/v) sucrose solution. To ensure optimal mating, twenty-five newly emerged females and thirty-three newly emerged males (maintaining a sex ratio of 1:1.3) were mixed in 30 cm × 30 cm × 30 cm cages made of gauze for a duration of three days (fig. S7).

The cotton plant, *Gossypium hirsutum*, was also reared at the Institute of Plant Protection, Chinese Academy of Agricultural Sciences. Briefly, the cotton seeds underwent an overnight soaking in water before being planted inside a glass greenhouse with a temperature of 26 ± 1 °C. Plants with eight true leaves were selected for experiments. These plants were then relocated to a climate chamber having a 14:10-hour light: dark photoperiod and a relative humidity of 65 ± 5 %.

**Gas stimulus preparation**

Three concentrations of CO_2_ (400, 1000, 10000, and100000 ppm) were prepared for the experiments. CO_2_-free synthetic air, comprising 21 % O_2_ and balanced N_2_, served both as the solvent for CO_2_ stimulations and as the control. Certified gas cylinders containing the gases were obtained from Beijing Shangtonghong Chemical (Beijing, China).

**Oviposition experiments in a semi-natural environment cage**

Oviposition experiments within a semi-natural environment included cultivated cotton plants placed inside a cage measuring 65 × 65 × 120 cm (length, width, and height). Six undamaged cotton plants were arranged in the cage so that the distances (left and right, front and back) between plants were approximately 16 cm and 22 cm. Sucrose solution (10 %) was placed in the middle of the cage to provide nutrients. Fifteen mated female moths were released into the cage (Fig. 1a). The cage, which housed 6 cotton plants and 15 mated *H*. *armigera* females, were positioned in a climate chamber illuminated, from 6:00 to 20:00, with artificial light. The moths were introduced into the cage 2 hours after the placement of the cotton plants. Moths were allowed to lay eggs on the plants for a period of three days. The number of eggs laid on all young versus old leaves of the same plant were counted. The oviposition index for each plant was calculated as (Egg number _young leaves_ - Egg number _old leaves_) / (Egg number _young leaves_ + Egg number _old leaves_). Four independent replications were performed, generating a total of 24 datasets.

**Observation of larval development**

The eggs were placed in plastic containers until hatching. Then ten newly emerged larvae were selected and put into a petri dish with young or old leaves of cotton (Fig. 1e). Every day, the young and old cotton leaves were replaced with fresh leaves of the two categories, until the tenth day. After five days, these larvae were placed individually in petri dish, avoiding the cannibalism. Every two days, the survival rate, body length, and body weight were recorded. The experiment was repeated nine times.

**Oviposition experiments in a semi-natural environment cage within an open-top chamber**

The abovementioned semi-natural environment cages, each housing 6 cotton plants and 15 mated *H*. *armigera* females, were situated within two open top chambers (OTCs). Each OTC was subjected to distinct environmental conditions: 1) control ambient CO_2_ (~400 ppm), and 2) elevated CO_2_ (~1000 ppm). The temperature in both OTCs was maintained at 25 ± 1°C, with a relative humidity of 60 ± 5 %. The control ambient CO_2_ was sourced from the air entering the OTC facilities, while the elevated CO_2_ was supplied from artificial CO_2_ gas tanks. Continuous monitoring and adjustment of CO_2_ concentrations in each OTC were performed using a CO_2_ analyzer (JUST ORIGIN, B3X; Shanghai, China) (Fig. 1i). Moths were allowed to lay eggs on the plants in the OTCs for a period of three days. The number of eggs laid on all young versus old leaves of the same plant were counted. The oviposition index was calculated as (Egg number _young leaves_ - Egg number _old leaves_) / (Egg number _young leaves_ + Egg number _old leaves_). Here, four independent replications were conducted, resulting in a total of 24 datasets.

**Measurement of CO_2_-emission from different parts of the cotton plant**

All measurements were conducted between 8:00 PM and 6:00 AM the subsequent day, during the natural period of darkness. The metabolic rate was assessed under two conditions: while on the plant (*ON plant*) and when detached from the plant (*OFF plant*).

The *ON plant* metabolic rate of both young and old leaves was assessed utilizing a commercial leaf photosynthesis system (LI-6400XT, LI-COR Inc., USA) (fig. S3a). Sixteen representative cotton plants were selected at the stage of eight true leaves. Measurements, including respiration rate (RR), intercellular CO_2_ pressure (Ci_Pa), intercellular CO_2_ concentration (Ci), and surface CO_2_ concentration (C2sfc), were conducted using the LI-6400 portable photosynthesis system equipped with a LED leaf chamber (2 cm × 3 cm).The *OFF plant* metabolic rate of young versus old leaves was determined using indirect calorimetry, wherein the CO_2_ production of excised leaves was measured with a Li-7000 CO_2_ analyzer (LI-COR) (fig. S3b). In total, twelve cotton plants, each possessing eight true leaves, were subjected to measurement. The flow outputs from the analyzer, the leaf chamber, and an A/D converter connected to a computer were established using Sable Systems equipment (Sable System International, SSI). The experimental setup involved sampling CO_2_ recordings from an empty chamber as a control, alongside two respirometry chambers. Each chamber measured the CO_2_ production of either an old leaf or several young leaves of the same weight.

Respirometry recordings were analyzed utilizing ExpeData PRO software (Sable Systems International, v1.8.4). Correction for CO_2_ lag time, due to the distance from the chamber to the analyzer, was calculated and subsequently subtracted from the CO_2_ value in each respirometry chamber. The absolute CO_2_ levels (ppm) were then exported to Excel, where metabolic output was aligned with activity. Statistical comparisons of means were conducted using a paired two-tailed Student's *t*-test with a significance level at 0.05.

**Y-tube olfactometer assay**

The effect of CO_2_ on the attraction behavior of *H*. *armigera* moths was investigated using a Y-tube olfactometer (with a stem and each arm measuring 10 cm, arms set at a 60° angle, and an internal diameter of 3 cm). CO_2_ and synthetic air were introduced into each of the olfactometer arms through Teflon tubing, secured in place by a rubber stopper. Airflow, maintained at 1 L/min per arm, was regulated using two flowmeters (LZB-3WB, China). The Y-tube experiments were carried out in dark conditions at 26 ± 1 °C, 65 ± 5 % relative humidity, and illuminated by red light for observation. Four-day-old males and females, both virgin and mated, were subjected to testing in the Y-tube olfactometer.

**Oviposition choice test**

Oviposition choice of mated female moths to CO_2_ was examined through a two-choice test in an oviposition cage. The box, constructed of acrylic, comprised a testing chamber with smooth surfaces measuring 60 cm (length) × 40 cm (width) × 75 cm (height), along with an exhaust shaft and an exhaust fan (Fig. 3b). To prevent moths from flying into the exhaust shaft, a 10 cm diameter metal gauze was inserted between the chamber and the exhaust shaft. Two small holes (1 cm diameter) at the bottom of the chamber allowed entry of the tested gases (CO_2_ and control) at a flow rate of 2.5 L/min. Yellow pipette tips were positioned around these holes, forming two circular areas with a diameter of 12 cm. During the oviposition choice test, these circular areas were covered by gauzes (15 cm × 15 cm), serving as an oviposition substrate.

To assess the responses of mated females under different concentrations of CO_2_, oviposition choice tests were conducted at three CO_2_ levels (400 ppm, 1000 ppm, and 10000 ppm). All experiments were carried out during the scotophase, at 26 ± 1 °C, and with a relative humidity of 65 ± 5 %. At the beginning of the experiment, fifteen mated females were placed into the testing chamber. After 10 hours, the number of eggs laid on the CO_2_ gauze and control gauze, respectively, were counted. Fifteen replicates were performed for each CO_2_ concentration. The oviposition index was computed as (Egg number _CO2 gauze_ - Egg number _control gauze_) / (Egg number _CO2 gauze_ + Egg number _control gauze_). Statistical comparisons of means were conducted using a paired two-tailed Student's *t*-test with a significance level at 0.05.

**CO_2_ receptor gene cloning**

The cDNA templates were generated from RNA samples extracted from the labial palps using the RevertAid First Strand cDNA Synthesis Kit (Thermo Fisher Scientific, USA). Specific primers were designed according to the full-length ORF sequences of *HarmGR1*, *HarmGR2*, and *HarmGR3*. The primer sequences are provided in Table S2. The successfully cloned GR ORF sequences were submitted to NCBI, and their respective GenBank accession numbers are OR734304, OR734305, and OR734306.

**Phylogenetic analysis**

Amino acid sequences of HarmGR1, HarmGR2, and HarmGR3 were employed for phylogenetic analysis along with 28 other CO_2_ receptor GRs from eleven species, including *D*. *melanogaster* (1, 2), *Musca*. *Domestica* (3), *Chrysomya*. *megacephala* (3), *Ceratitis*. *capitata* (4), *Anopheles*. *coluzzii* (4), *A*. *sinensis* (4), *Aedes*. *aegypti* (5), *Culex*. *Quinquefasciatus* (4), *Bombyx*. *Mori* (6), *Triboblium*. *Castaneum* (7), and *Dendroctonus*. *Ponderosae* (8). A phylogenetic tree was constructed using RAxML with the Jones-Taylor-Thornton (JTT) amino acid substitution model, and node support was assessed with 1000 bootstrap replicates. FigTree v1.4.4 was utilized for enhancing visualization of the phylogenetic tree.

**Quantitative real-time PCR analysis**

Total RNA was extracted from the head, thorax, abdomen, legs, proboscis, antenna, labial palps, and genital gland of females and males using TRIzol. First-strand cDNA was synthesized with RevertAid first-strand cDNA synthesis kit (Thermo Scientific, Waltham, MA, United States). qRT-PCR was carried out using GoTaq® qPCR Master Mix (Promega, WI, United States) in an ABI 7500 Real-Time PCR System (ABI, Vernon, CA, United States). *HarmActin* served as a reference gene, and the amplification efficiency of each primer pair was confirmed through a melting curve analysis. The primers are listed in Table S2. Relative gene expression levels were calculated using the 2^-ΔΔCT^ method. Differences in expression across tissues and between sexes were analyzed by one-way ANOVA, followed by Duncan's test (*P* < 0.05).

**CRISPR-Cas9-based genome editing and construction of homozygote mutants**

Genome editing of *HarmGR1*, *HarmGR2*, and *HarmGR3* were performed following established protocols (9). First, target sites for single guide RNAs (sgRNAs) were selected on the exons of three *HarmGRs* based on the principle of manufacturer’s instructions (GeneArt™ Precision gRNA Synthesis Kit, ThermoFisher Scientific). For *HarmGR1*, a sgRNA target site (5’-CTAAGAGAACCACTTTCAATTGG-3’) on exon 2 was identified. Two target sites for sgRNAs (5’-CTAAGAGAACCACTTTCAATTGG-3’ and 5’-GCTCAGTCTCTGTATACTCATGG-3’) were designed on the third and fourth exon of *HarmGR2*. Two sites for sgRNAs (5’-CAAGAAAGCACTAGCCATGGCGG-3’ and 5’-CTGCGCTAATATCTTGGCTGAGG-3’), located in the second and third exon, were selected to be target for *HarmGR3*. After that, these sgRNAs were prepared by PCR assembly, following the manufacturer’s instructions (GeneArt™ Precision gRNA Synthesis Kit, ThermoFisher Scientific). The DNA template of each sgRNA, containing a specific T7 promoter and the guide RNA sequence, was generated using synthetic forward and reverse oligonucleotides with the Tracr Fragment + T7 Primer Mix (Table S2). The Cas9 protein used here was obtained from ThermoFisher (GeneArt Platinum Cas9 Nuclease, ThermoFisher Scientific, Pittsburgh, PA).

The prepared sgRNA and Cas9 protein were co-injected into freshly laid eggs. These injections were performed within an hour after oviposition using a FemtoJet and injectMan NI 2 microinjection system (Eppendorf, Hamburg, Germany). Approximately 500 eggs were injected for each *HarmGR* gene, and the injected eggs were then incubated at 27 ± 1 °C and 65 ± 5 % RH for 3-4 days until hatching.

Female and male adults of the G0 generation from the microinjected eggs were crossbred with WT moths to generate F1 individuals. Upon the emergence of F1 moths, two types of mutant identification were performed for *HarmGR1*, *HarmGR2*, and *HarmGR3*, respectively. Specifically, for *HarmGR1*, pairs of randomly chosen F1 moths were placed in plastic cups for mating and egg-laying. After oviposition, genomic DNA was extracted from their mid legs, and PCR reactions were conducted to amplify an 855-bp fragment containing the target site, using gene-specific primers (Table S2). To confirm genotypes, the PCR products were purified, sub-cloned into pEASY Blunt cloning vectors (TransGen, Beijing, China), and then subjected to sequencing. F1 moths with heterozygous mutants, exhibiting a 1-bp deletion at exon 3, were selected and subjected to mass crossing to generate F2. Homozygous mutants in F2 were identified through PCR sample sequencing and utilized in subsequent experiments. For *HarmGR2* and *HarmGR3* mutations, genomic DNA was extracted from each F1 adult using a middle leg and the TIANamp Genomic DNA Kit (Tiangen, China). Afterwards, the genomic DNA served as a template to amplify a fragment containing the target site with specific primers (Table S2). The PCR-amplified fragment was employed for genotyping mutants through gel detection and sequence confirmation. The screened F1 moths were crossed with each other to produce offspring (F2). After the emergence of F2 moths, their middle legs were utilized to extract genomic DNA for further genotyping mutants through gel detection.

**Electrolabialpalpography (ELPG) and Electroantennography (EAG) recording**

The ELPG experiment was conducted on 3-4 days old individuals, following previously described procedures (10). Each labial palp of male and female moths was obtained by cutting at its base. In each test, CO_2_ stimulations at different concentrations were sequentially delivered using a self-regulating stimulus flow controller, with synthetic air used as a control. Each stimulation was applied through 1-second pulses at a flow rate of 0.8 L/min. The ELPG signals were recorded, monitored, and analyzed using Syntech EAG-software (Syntech, Germany). The ELPG response values for each stimulation were calculated by subtracting the value of the same labial palp corresponding to control (CO_2_-free synthetic air). A minimum of thirty replicates were performed for each concentration of CO_2_.

The EAG experiments were also carried out in 3-4 days old females and males. The antennae were cut at the base of the flagellum and inserted between two glass electrodes filled with 0.1 M KCl solution. The CO_2_ stimuli were applied by a self-regulating stimulus flow controller as mentioned in ELPG experiments. EAG data were recorded and analyzed as for the method used in ELPG recording.

**Single sensilla recording**

*H*. *armigera* females, aged three to four days post emergence, were immobilized within 1 mL plastic pipette tips. By using a stereomicroscope (SZ61, Olympus, Germany), the protruding head, antennae, proboscis, and other organs were securely fixed to the edge of the pipette tip with dental wax, leaving only one of the labial palps accessible. The exposed labial palp was then immobilized with dental wax, leaving the tip containing the opening of the LPO accessible. A silver wire serving as reference electrode was carefully inserted into one of the moth's compound eyes, while a sharp quartz recording electrode was vertically inserted into the LPO. Spikes were recorded when a stable electric signal was achieved. Electrophysiological signals were amplified through an Axoclamp 900A amplifier and displayed on a computer screen using the software package Autospike 2 8.01 (Syntech, Germany).

To deliver CO_2_ stimulations, we employed a self-regulating stimulus flow controller, as detailed in our prior research work (10). A consistent flow of purified and humidified CO_2_-free synthetic air was directed onto the opening of the LPO through the outlet of a 14-cm-long metal tube. CO_2_ stimulus pulses at concentrations of 1000 ppm, 10000 ppm, and 100000 ppm were sequentially administered, each for a duration of 5 seconds.

**Calcium imaging**

Three-to-four-day-old moths were used in calcium imaging measurements. A membrane permeable fluorescent calcium indicator (Calcium Green-1 acetoxymethyl (AM), Thermo), mixed with 5 % Pluronic F-127 (Thermo) and dissolved in physiological saline solution, was added to the exposed brain for bath application. The imaging setup involved a CCD camera (Olympus U-CMAD3) mounted on an upright microscope (Olympus BX51Wl), which was equipped with a water immersion objective (Olympus, 10x/0.30). The excitation of Calcium green was achieved at 475 nm.

Each moth was gently immobilized, preserving its labial palps and antennae in an intact state. Following the opening of the head capsule between the compound eyes, the tissue covering the brain was carefully removed to expose the antennal lobe. To facilitate imaging, Calcium green solution at 4 °C was applied onto the antennal lobe 1 hour before the recording started. After eliminating excess dye, the brain was placed frontally under the microscope for observing the CO_2_ glomerulus, LPOG. Each trial had a duration of 10 s and was recorded with a sampling rate of 4 Hz, resulting in 40 frames. The intertrial interval was at least 1 min, to prevent potential adaptation. The stimulation settings used in this experiment, including stimulation window, CO_2_ concentrations, and stimulus sequence, were identical to those employed in single sensilla recording experiments.

The calcium imaging data were analyzed using ImageJ. First, bleach correction and a Gaussian blur filter were applied to improve the signal-to-noise ratio of the data. Subsequently, the baseline fluorescence (F0) was determined by averaging the intensities of the first 6 frames. Finally, the images depicting response of antennal lobe glomeruli were generated using the deltaF/F0 function.

References

1. Jones WD, Cayirlioglu P, Kadow IG *et al*. Two chemosensory receptors together mediate carbon dioxide detection in *Drosophila*. *Nature* 2007; **445**: 86-9.

2. Kwon JY, Dahanukar A, Weiss LA *et al*. The molecular basis of CO_2_ reception in *Drosophila*. *Proc Natl Acad Sci U.S.A.* 2007; **104**: 1741-1751.

3. Xu W and Anderson A. Carbon dioxide receptor genes in cotton bollworm *Helicoverpa armigera*. *Sci Nat* 2015; **102**: 11.

4. Coutinho-Abreu IV, Sharma K, Cui L *et al*. Odorant ligands for the CO_2_ receptor in two Anopheles vectors of malaria. *Sci Rep* 2019; **9**: 2549.

5. Kent LB, Walden KK, Robertson HM. The Gr family of candidate gustatory and olfactory receptors in the yellow-fever mosquito *Aedes aegypti*. *Chem Senses* 2008; **33**: 79-93.

6. Liu NY, Xu W, Papanicolaou A *et al*. Identification and characterization of three chemosensory receptor families in the cotton bollworm *Helicoverpa armigera*. *BMC Genomics* 2014; **15**: 597.

7. Robertson HM and Kent LB. Evolution of the gene lineage encoding the carbon dioxide receptor in insects. *J Insect Sci* 2009; **9**: 19.

8. Andersson MN, Keeling CI, Mitchell RF. Genomic content of chemosensory genes correlates with host range in wood-boring beetles (*Dendroctonus ponderosae*, *Agrilus planipennis*, and *Anoplophora glabripennis*). *BMC Genomics* 2019; **20**: 690.

9. Guo M, Du L, Chen Q *et al*. Odorant receptors for detecting flowering plant cues are functionally conserved across moths and butterflies. *Mol Biol Evol* 2021; **38**: 1413-1427.

10. Chen Q, Liu X, Cao S *et al*. Fine structure and olfactory reception of the labial palps of *Spodoptera frugiperda*. *Front Physiol* 2021; **12**: 680697.

**
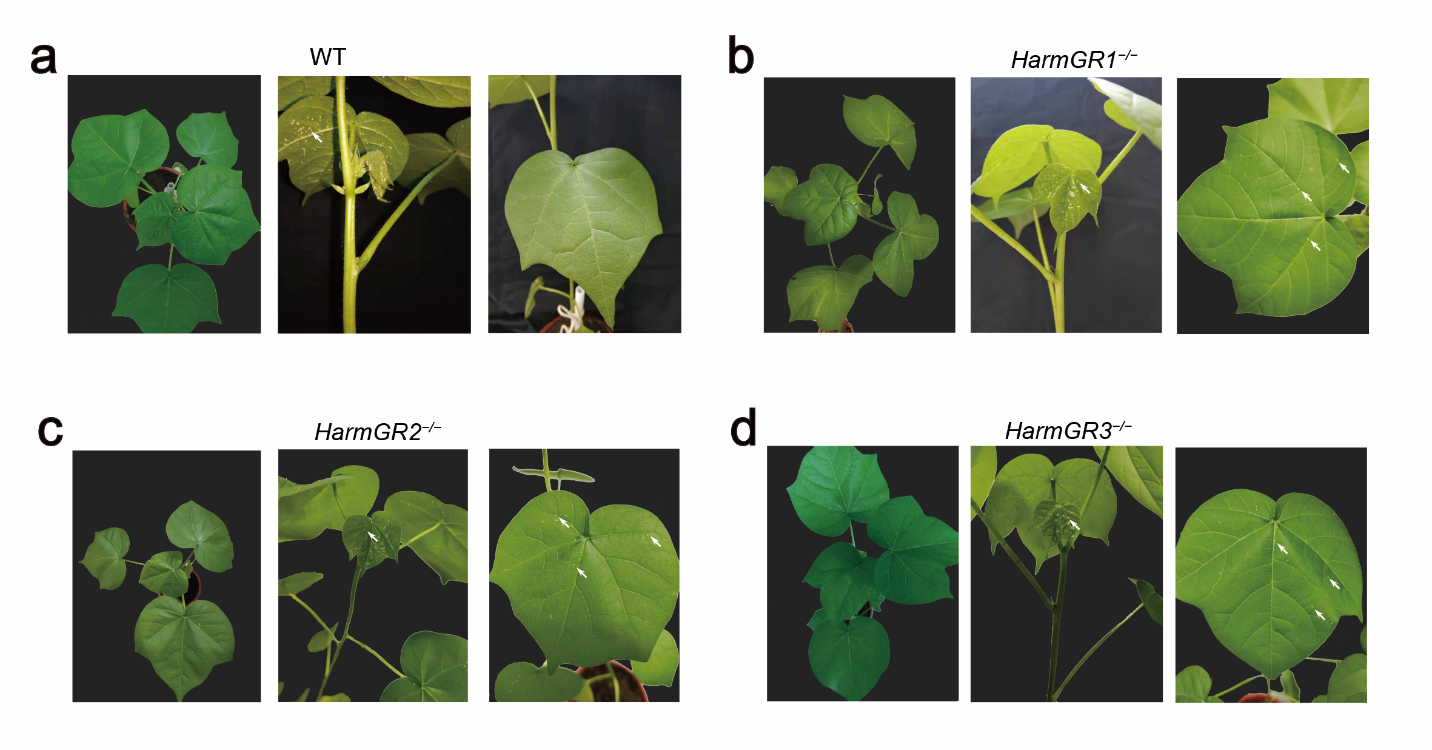
Fig. S1.** Photos showing eggs laid by wild-type and mutant *H. armigera* moths on cotton plants in a seminatural environment (related to Fig. 1 and Fig. 5). (a-d) Cotton plants with eggs laid by wild-type (a), by *HarmGR1^−/−^* mutants (b), by *HarmGR2^−/−^* mutants (c), and by *HarmGR3^−/−^* mutants (d), *left*, top view; *middle,* young leaves; *right*, old leaves. Eggs laid on the leaves were marked by *white* arrows.

**
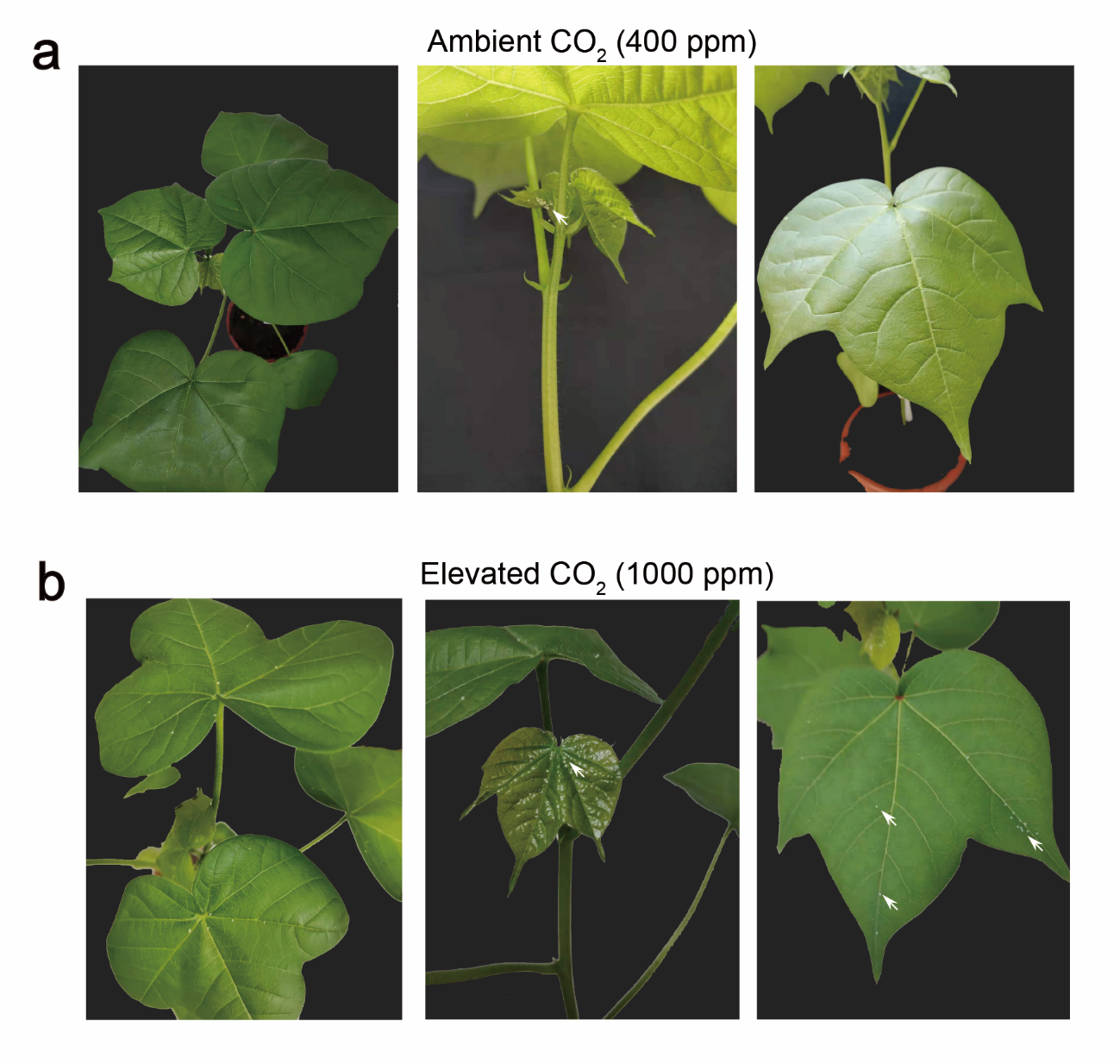
**

**Fig. S2.** Photos demonstrating oviposition in two OTCs possessing different CO_2_ concentrations (Related to Fig. 1). (a and b) Photos of eggs laid by *H. armigera* in the context of current ambient CO_2_ (400 ppm, a), and in elevated CO_2_ (1000 ppm, b). *left*, top view; *middle,* young leaves; *right*, old leaves. Eggs laid on the leaves were marked by *white* arrows.


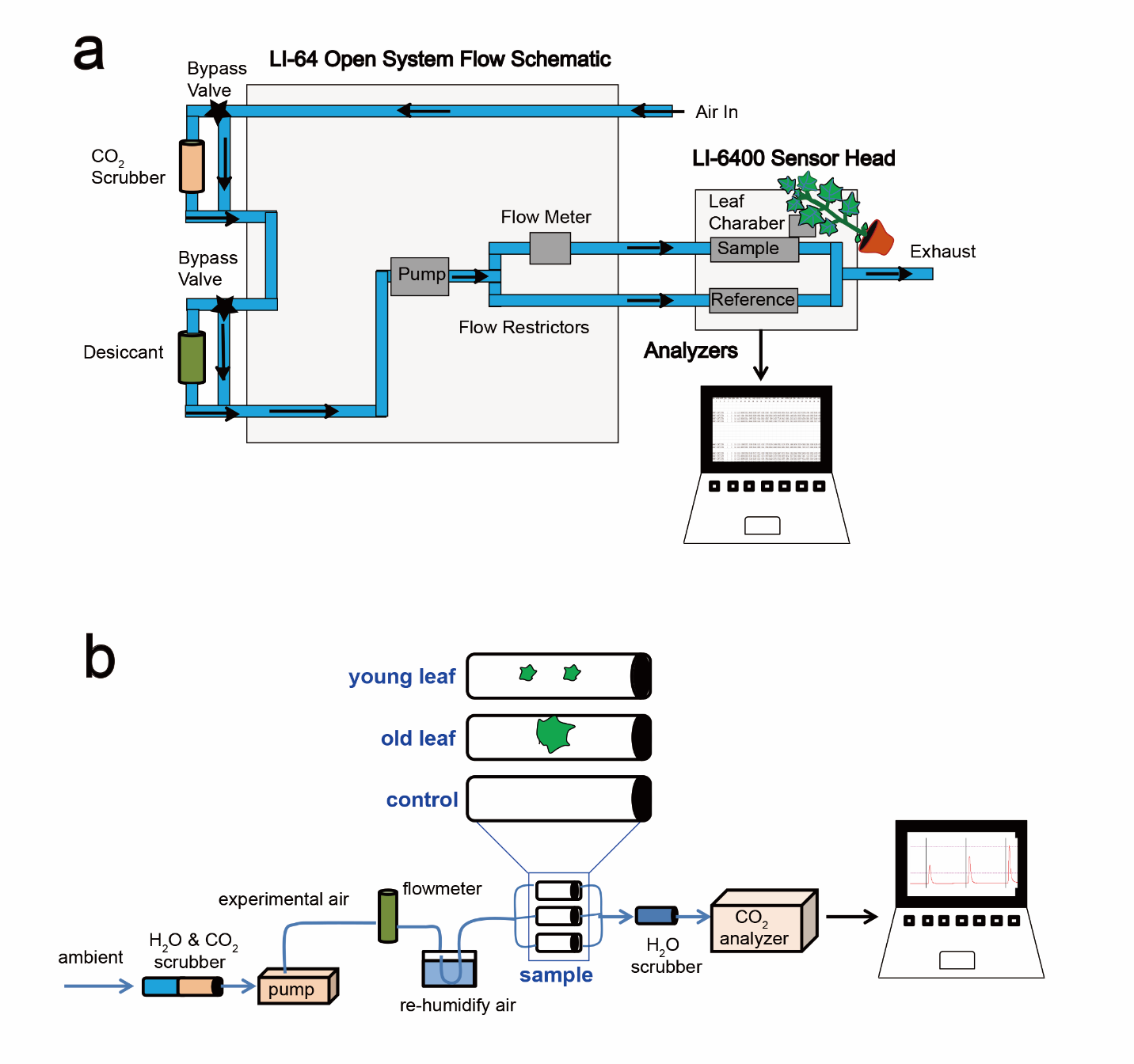


**Fig. S3.** Schematic drawing of the system to measure metabolic rate in the young and old leaves. (related to Fig. 2). (a) The *ON plant* metabolic rate of both young and old leaves was assessed utilizing a commercial leaf photosynthesis system (LI-6400XT, LI-COR Inc., USA). (b) The *OFF plant* metabolic rate of young versus old leaves was determined using indirect calorimetry, wherein the CO_2_ production of excised leaves was measured with a Li-7000 CO_2_ analyzer (LI-COR).


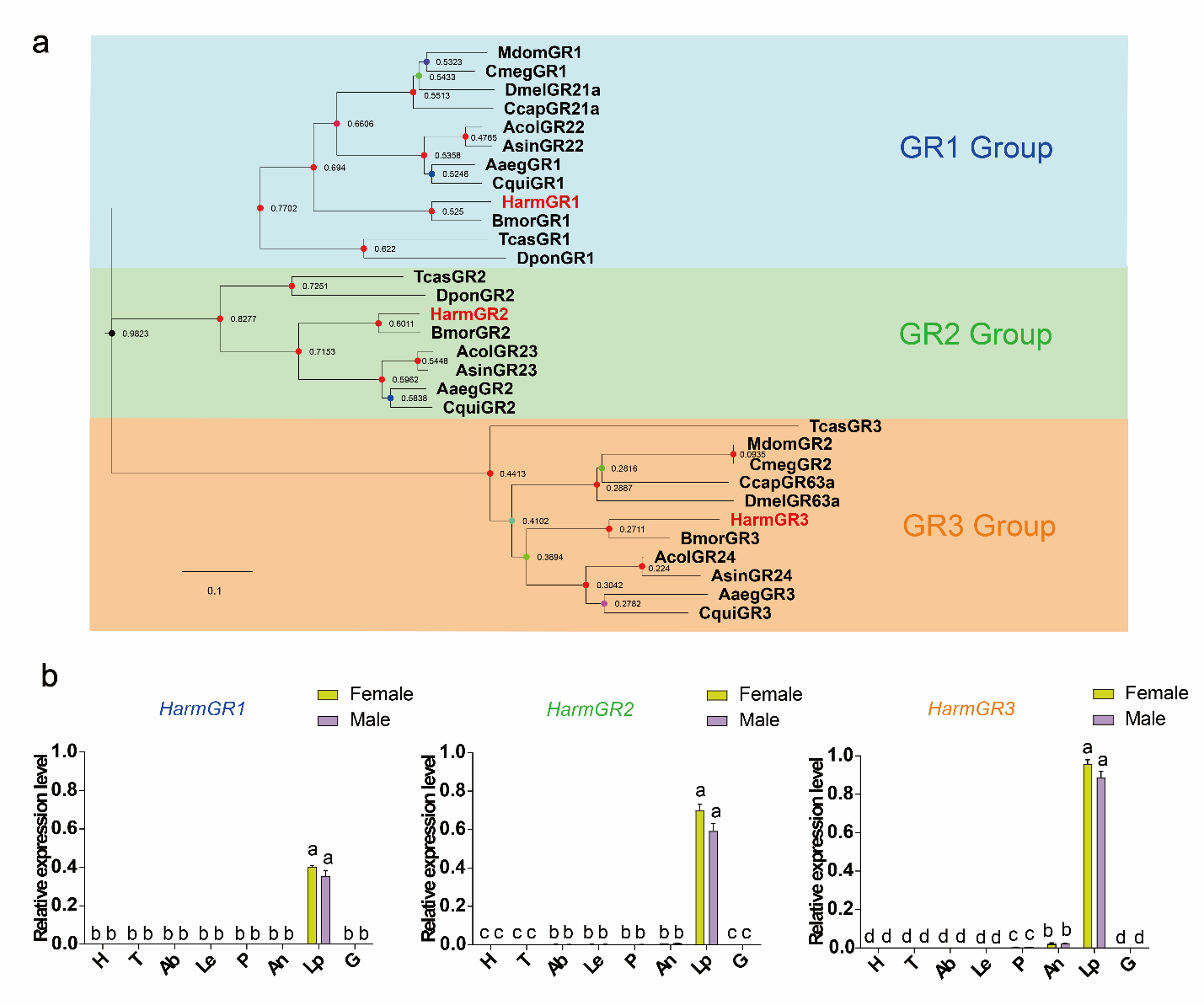


**Fig. S4.** Phylogenetic analysis and expression pattern of the candidate CO_2_ receptors in *H.armigera* (Related to Fig. 4). (a) Phylogenetic tree of 31 CO_2_ receptors in 12 insect species. The 12 insect species, included *D. melanogaster*, *M. domestica*, *C. megacephala*, *C. capitata*, *A. coluzzii*, *A. sinensis*, *A. aegypti*, *C. quinquefasciatus*, *H. armigera*, *B. mori*, *T. castaneum*, *D. ponderosae*. (b) Quantitative analysis of relative expression of *HarmGR1*, *HarmGR2,* and *HarmGR3* across different tissues or organs in *H. armigera*. H: head; T: thorax; Ab: abdomen; Le: legs; P: proboscis; An: antenna; Lp: labial palps; G: genital gland. Bar plots illustrated as mean ± SEM. Statistical significance, denoted by lowercase letters, was observed at a threshold of *p* < 0.05 (Duncan^’^s multiple range test, n = 3).


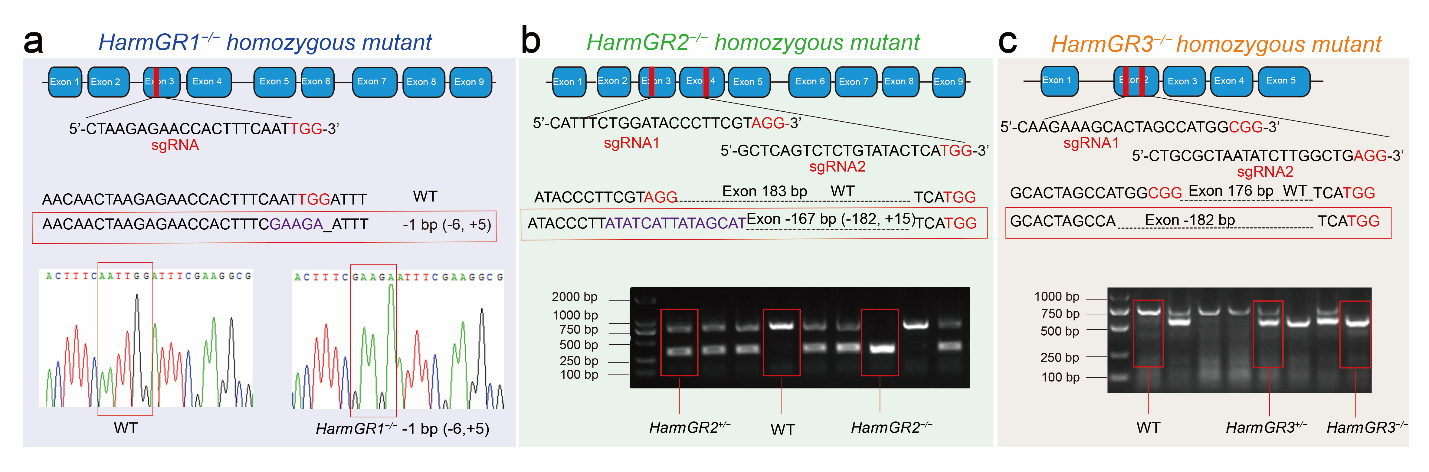


**Fig. S5.** CRISPR/Cas9-based *HarmGR* genes knockout (Related to Fig. 4). (a) Knockout of the *HarmGR1* gene and the screening of *HarmGR1* mutants. Top: schematic diagram of the sgRNA with target sites (*black*) and PAM sequences (*red*). Middle: The strains of wild-type and *HarmGR1^−/−^* mutant (*red* frame) in the adult of F1 generation with PAM sequences in *red*, and the insertion bases in *purple*. Bottom: the two sequencing chromatograms indicated wild-type (left) and *HarmGR1^−/−^* homozygous mutation 1 (right), respectively, where the mutation site is shown in a *red* frame. (b and c) Knockout of the *HarmGR2* (b) and *HarmGR3* (c) genes and screening of the corresponding mutants. Top: schematic diagram of the sgRNA with target sites (*black*) and PAM sequences (*red*). Middle: The strains of wild-type and the corresponding mutant (*red* frame) in the adult of F1 generation with PAM sequences in *red*, deleted bases replaced by dashed lines, and the insertion bases in *purple*. Bottom: the identification of genotypes of wild-type, heterozygote, and homozygote in individuals by PCR and agarose gel electrophoresis.


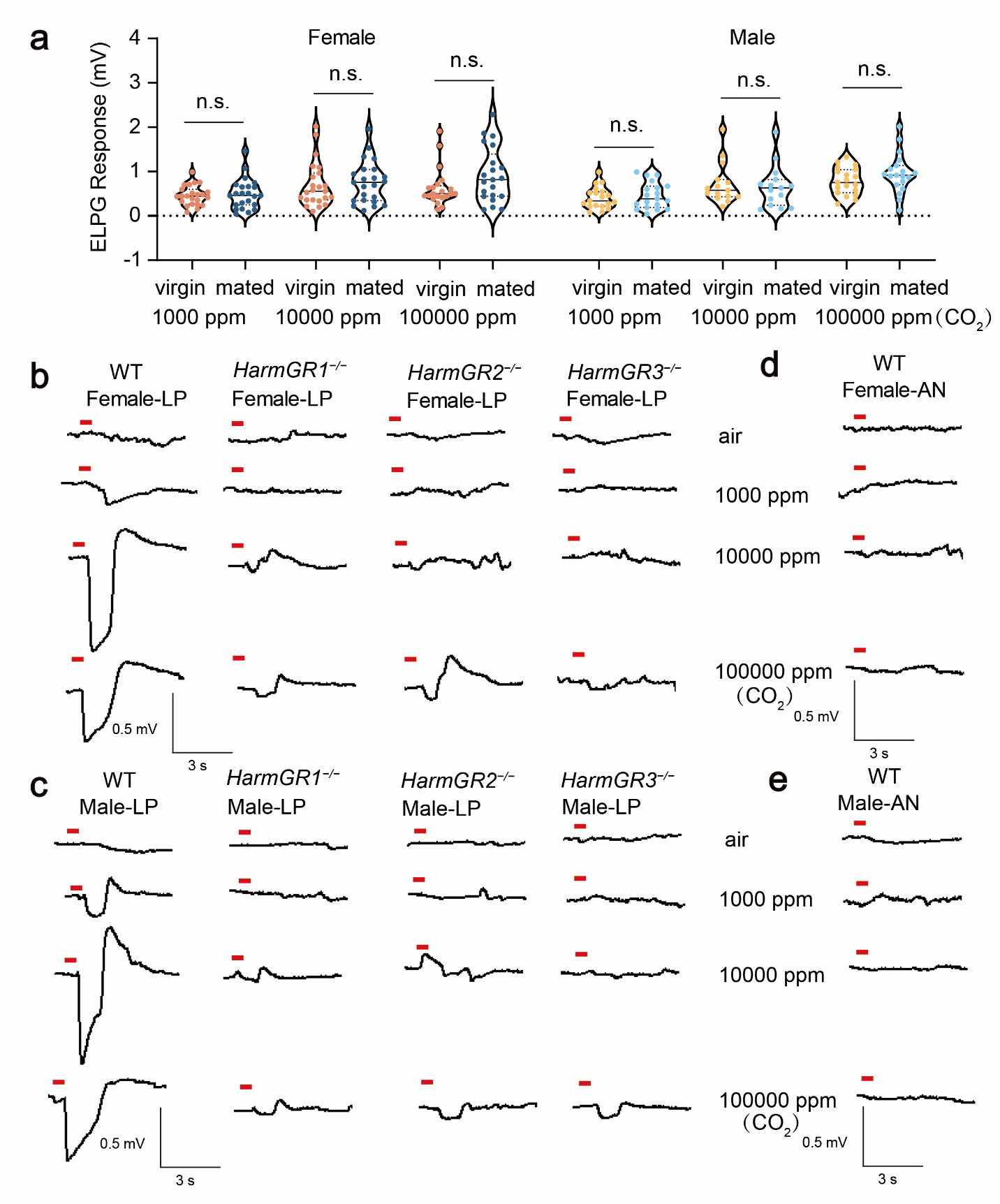


**Fig. S6.** ELPG data in virgin and mated moths, and examples of ELPG and EAG data in wild-type and *HarmGR* mutants during exposure to CO_2_ (Related to Fig. 4). (a) ELPG responses to CO_2_ in virgin and mated *H*. *armigera*. Data are presented as median with quartiles, with violin plots showing kernel density estimation., with n.s. indicating *p* > 0.05 (unpaired two-tailed Student’s *t*-test, n = 15-23). (b and c) ELPG traces in females (b) and males (c) in four strains, including wild-type, *HarmGR1^−/−^*, *HarmGR2^−/−^* and *HarmGR3^−/−^* mutants. (d and e) EAG traces of female (d) and male (e) wild-type *H*. *armigera.* AN, antenna; LP, labial palp.


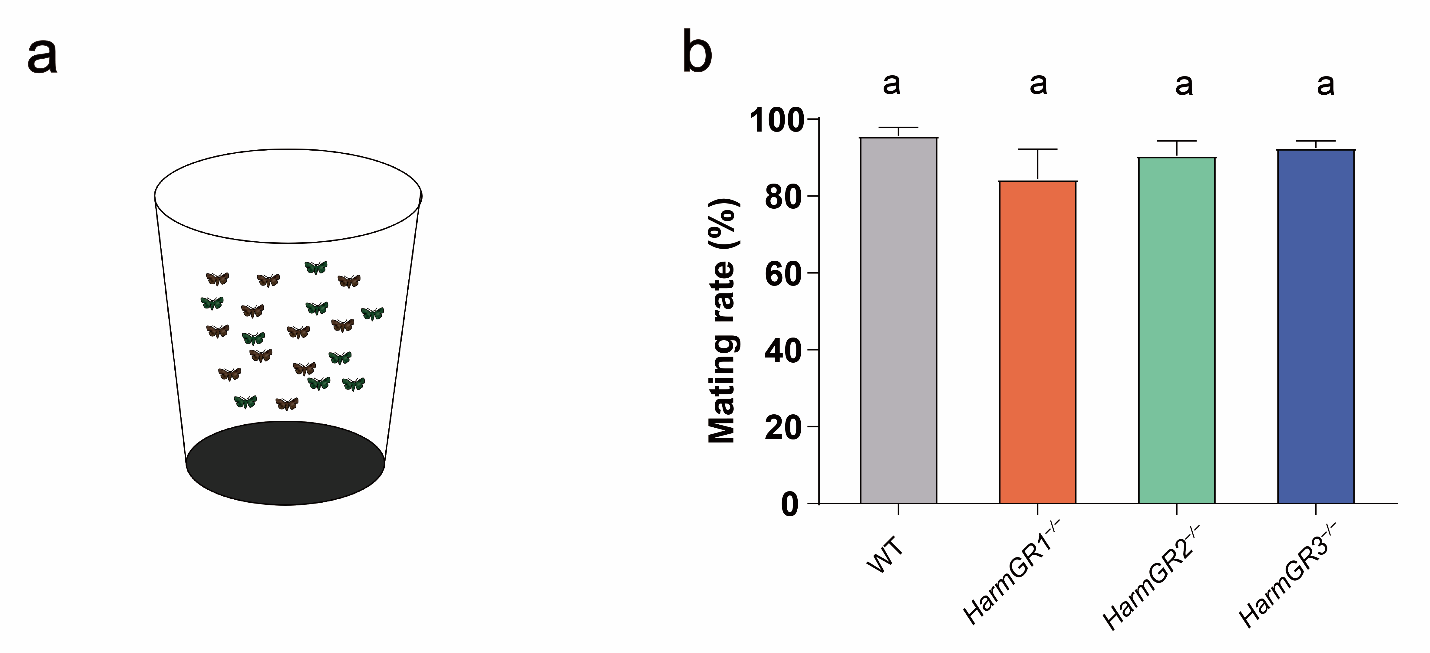


**Fig. S7.** Screening of female mating success before oviposition experiments (Related to Figure 1, 3, 5). (a) A mating container for *H. armigera* copulation, with a female - male ratio of 1:1.3. (b) Indication of mating rate in both wild type and mutant females based on ovary dissection. Bar plots illustrated as mean ± SEM. Statistical significance, denoted by lowercase letters, was observed at a threshold of *p* < 0.05 (Duncan^’^s multiple range test, n = 5).

Table S1.

The Concrete values of *ON plant* metabolic rate of both young and old leaves. Related to Fig. 2, A-D

|  | **Young leaves** | **Old leaves** | ***P*** |
| --- | --- | --- | --- |
| Respiration rate (µmol CO_2_ m^-2^s^-1^) | 1.41 ± 0.08 | 0.46 ± 0.03 | < 0.001 |
| Intercellular CO_2_ pressure (Pa) | 73.86 ± 3.61 | 56.98 ± 1.87 | < 0.001 |
| Intercellular CO_2_ concentration (ppm) | 754.90 ± 47.20 | 553.14 ± 15.00 | < 0.001 |
| Surface CO_2_ concentration (ppm) | 658.68 ± 17.64 | 475.02 ± 6.43 | < 0.001 |

Data showed in mean ± SEM.

Table S2.

Primers used in this study. Related to Methods.

| **Primer name** | **Sequence (5'-3')** |
| --- | --- |
| ***Primer for gene cloning*** | |
| HarmGR1-F | ATGAACAAAGAAGAACATGGTTT |
| HarmGR1-R | ATTCTTCATCAACGTCATCAGTA |
| HarmGR2-F | ATGACGATCCCGGATCATCTGTTT |
| HarmGR2-R | TACGTTGCAACAATTTGTGGGTCC |
| HarmGR3-F | ATGTCGTTTCATACCAGTAA |
| HarmGR3-R | TGTTACTGGTATGAAACGACAT |
| ***Primer for qRT-PCR*** | |
| HarmGR1-F | CGCGAAGGATCTTTATGGTCCG |
| HarmGR1-R | AGCACAAGCAAACTCTTCGTGG |
| HarmGR2-F | TACTCATGGATGGATTCCTGCTGAG |
| HarmGR2-R | TACCATAGGGCACAGTTCATGTTGA |
| HarmGR3-F | AAGGACATTGGTCTTGCATTGACTG |
| HarmGR3-R | CCACCATTAGCAGCTTCTTTTGGAA |
| HarmActin-F | GGCATGGGGCAGAAGGACTC |
| HarmActin-R | ATGATGCCGTGCTCGATGGG |
| ***Primer for sgRNA synthesis*** | |
| HarmGR1-F | TAATACGACTCACTATAGCTAAGAGAACCACTTT |
| HarmGR1-R | TTCTAGCTCTAAAACATTGAAAGTGGTTCTCTT |
| HarmGR2-F1 | TAATACGACTCACTATAGATTTCTGGATACCCT |
| HarmGR2-R1 | TTCTAGCTCTAAAACACGAAGGGTATCCAGAAA |
| HarmGR2-F2 | TAATACGACTCACTATAGCTCAGTCTCTGTATA |
| HarmGR2-R2 | TTCTAGCTCTAAAACTGAGTATACAGAGACTGA |
| HarmGR3-F1 | TAATACGACTCACTATAGAAGAAAGCACTAGCC |
| HarmGR3-R1 | TTCTAGCTCTAAAACCCATGGCTAGTGCTTTCT |
| HarmGR3-F2 | TAATACGACTCACTATAGTGCGCTAATATCTTG |
| HarmGR3-R2 | TTCTAGCTCTAAAACCAGCCAAGATATTAGCGC |
| ***Primer for genomic amplification of mutant sites*** | |
| HarmGR1-F | TCTTTATGGTCCGGAGATAACTGAT |
| HarmGR1-R | ACTTAGTATGGAGCTTGGAATGCAT |
| HarmGR2-F | GCACAATTACCTTCAGCTGGAGAT |
| HarmGR2-R | CTGCGAAAACATTCAGACAGGCTT |
| HarmGR3-F | CTACAAGCAGCTATCAGGCCGAA |
| HarmGR3-R | ACGCATAATGAGCTTCATCACAA |

The T7 promoter sequence is shown in red.
